# Verification of Intraovum Transmission of a Microsporidium of Vertebrates: *Pseudoloma neurophilia* Infecting the Zebrafish, *Danio rerio*

**DOI:** 10.1371/journal.pone.0076064

**Published:** 2013-09-23

**Authors:** Justin L. Sanders, Virginia Watral, Keri Clarkson, Michael L. Kent

**Affiliations:** 1 Department of Microbiology, Oregon State University, Corvallis, Oregon, United States of America; 2 Department of Biomedical Sciences, Oregon State University, Corvallis, Oregon, United States of America; Technion-Israel Institute of Technology Haifa 32000 Israel, Israel

## Abstract

Direct transmission from parents to offspring, referred to as vertical transmission, occurs within essentially all major groups of pathogens. Several microsporidia (Phylum Microsporidia) that infect arthropods employ this mode of transmission, and various lines of evidence have suggested this might occur with certain fish microsporidia. The microsporidium, *Pseudoloma neurophilia*, is a common pathogen of the laboratory zebrafish, *Danio rerio*. We previously verified that this parasite is easily transmitted horizontally, but previous studies also indicated that maternal transmission occurs. We report here direct observation of *Pseudoloma neurophilia* in the progeny of infected zebrafish that were reared in isolation, including microscopic visualization of the parasite in all major stages of development. Histological examination of larval fish reared in isolation from a group spawn showed microsporidian spores in the resorbing yolk sac of a fish. Infections were also observed in three of 36 juvenile fish. Eggs from a second group spawn of 30 infected fish were examined using a stereomicroscope and the infection was observed from 4 to 48 hours post-fertilization in two embryos. Intraovum infections were detected in embryos from 4 of 27 pairs of infected fish that were spawned based on qPCR detection of *P. neurophilia* DNA. The prevalence of intraovum infections from the four spawns containing infected embryos was low (∼1%) based on calculation of prevalence using a maximum likelihood analysis for pooled samples. Parasite DNA was detected in the water following spawning of 11 of the infected pairs, suggesting there was also potential for extraovum transmission in these spawning events. Our study represents the first direct observation of vertical transmission within a developing embryo of a microsporidian parasite in a vertebrate. The low prevalence of vertical transmission in embryos is consistent with observations of some other fish pathogens that are also readily transmitted by both vertical and horizontal routes.

## Introduction

Pathogens employ a range of different mechanisms to infect new hosts. Vertical or maternal transmission is characterized by pathogens being transmitted to progeny from parents, usually through an infected female host. This mode of transmission is employed by some obligate parasites, which cannot complete their life cycles without a host. The methods by which pathogens are transmitted can exert a powerful influence on virtually all aspects of the biology of the host, either directly or indirectly. Indeed, it was argued that pathogens and their effects on hosts are responsible for nearly all aspects of biological organization [1]. The population structure of the host species has an important influence on mode of transmission employed by pathogens [1], [2]. For example, in populations where individuals are spatially separated, minimizing the opportunity for horizontal transfer, selection would favor maternal, or vertical, transmission, ensuring propagation of the parasite.

The mode of transmission also has a direct influence on the virulence of a parasite with selection favoring levels of parasite reproduction, and thus virulence, that provide the highest level of fitness for the parasite. Pathogens which are transmitted vertically are generally less virulent that those which are transmitted horizontally as there is selective pressure for the survival of the infected female to live to reproductive age and pass the infection on to progeny [3]. As a result, theses parasites generally do not proliferate to high numbers in the host, minimizing disease and thus host mortality. The virulence-related characteristics of vertically transmitted pathogens are generally limited to those that increase the number of susceptible hosts, including feminization of male hosts [4].

Microsporidia are obligate intracellular pathogens with species infecting virtually all animal phyla. This distinctive group has undergone numerous taxomonic revisions since first being described [5]. Originally assigned to the Schizomycetes, a group containing yeast-like fungi [6], the Microsporidia were subsequently moved to other groups such as the Sporozoa [7] and Archaezoa [8]. Their assignment to the Archaezoa, a group of protists considered to be “primitive” due to the absence of some typical eukaryotic features, was based mainly on their apparent lack of mitochondria and was further supported by early molecular phylogenetic analyses based on small subunit ribosomal RNA gene sequences. This was, however, later found to be in error as subsequent molecular and ultrastructural analyses have shown the presence of relictual mitochondria [9] and more sophisticated phylogenetic analyses that account for rate variation and the presence of numerous fungal-type products, notably trehalose and chitin, placed the Microsporidia once again among the Fungi. Their exact placement among the Fungi (i.e., as early-branching or sister to the Fungi) is currently debated [10].

Microsporidia have been shown to employ a diverse range of transmission strategies. Several microsporidian species are transmitted horizontally, generally by ingestion of spores from either water contaminated by feces or tissue from dead infected hosts[11]. Maternal transmission of microsporidian parasites has been noted for several species infecting crustaceans [12] and insects [13]. Whereas some microsporidian species are only vertically transmitted [14], many are transmitted both horizontally and vertically. Maternal transmission occurs by two general mechanisms, which in arthropods are characterized as transovarial (within the egg) and transovum (parasite is shed outside of the egg during egg laying/spawning). These terms were developed to describe vertically transmitted microsporidia of insects, and are somewhat confusing when applied to vertebrates. Hence, we refer to these two types of transmission as intraovum and extraovum, respectively.

Regarding vertebrates, the earliest recorded evidence of vertical transmission comes from Hunt et al [15] who observed *Encephalitozoon cuniculi* infections in gnotobiotic rabbits, strongly suggesting transplacental transmission of this parasite. Also, several lines of indirect evidence support vertical transmission of microsporidia in fishes. The strongest evidence of vertical transmission in fish comes from *Ovipleistophora ovariae*, a microsporidium that infects the golden shiner, *Notemigonus crysoleucas*, and has been observed to infect only female fish. In this species, spores are found almost exclusively within the ovaries and developing oocytes [16]. While large amounts of *O. ovariae* DNA has been detected within surface-decontaminated, spawned eggs and developing larval fish [17], the parasite has not been directly observed in the progeny of infected fish. *Ovipleistophora mirandellae* also infects the oocytes and eggs of cyprinid fishes [18] and thus has been suggested [19] to undergo intraovum transmission. *Loma salmonae* is a microsporidian pathogen of salmon and trout, and has been observed in the ovigerous stroma, but not eggs [20]. The translocation of *Loma salmonae* to fish farms in Chile which had been populated using only surface decontaminated, fertilized eggs is also suggestive of vertical transmission [20], [21].

The zebrafish, *Danio rerio*, is an important laboratory model for toxicology, developmental biology, cancer, and infectious disease research. It has a well-characterized immune system [22] and the complete genome has been sequenced with the genetic map showing an overall highly conserved synteny with the human genome [23]. A microsporidium, *Pseudoloma neurophilia*, is responsible for chronic infections of zebrafish [24], [25] and is common in zebrafish research colonies [26]. The high prevalence of a microsporidium in laboratory zebrafish populations provides an ideal opportunity to learn more about the transmission characteristics of this parasite. As the name implies, *Pseudoloma neurophilia* chronically infects neural tissue and develops into mature spores mainly in the hindbrain, spinal cord, motor nerve ganglia and spinal nerve roots. The parasite enters the host via the intestinal epithelium and infects extraintestinal skeletal muscle myocytes in early stages of the infection [27]. Infections by the parasite result in a spectrum of disease ranging from minimal to no clinical presentation to acute mortality. We have shown that *P. neurophilia* is horizontally transmissible by bath exposure and cohabitation with live fish, ostensibly *per os*. There is growing evidence of vertical transmission for this parasite as follows: 1. The presence of microsporidian spores visualized by histology in the ovaries and developing oocytes of infected adult female fish [28], 2. *P. neurophilia* DNA detected by PCR in spawn water and eggs from infected adults [29], 3. High numbers of *P. neurophilia* spores in ovaries of infected females [25], 4. Repeated detection of *P. neurophilia* in the progeny of two lines of zebrafish screened as part of a protocol to develop a specific pathogen free colony leading to the exclusion of these lines from the colony [30].

Despite the significant indirect evidence, observation of the intra-ovum transmission of a microsporidian parasite in a vertebrate has not been verified. By utilizing a combination of a qPCR method [29] and microscopy on a number of spawning fish and progeny, we provide direct evidence of the intraovum transmission of *P. neurophilia*. Additionally, we provide data supporting the hypothesis that extraovum maternal transmission is also an important route of transmission.

## Results

### Group spawns

**Figure 1:**
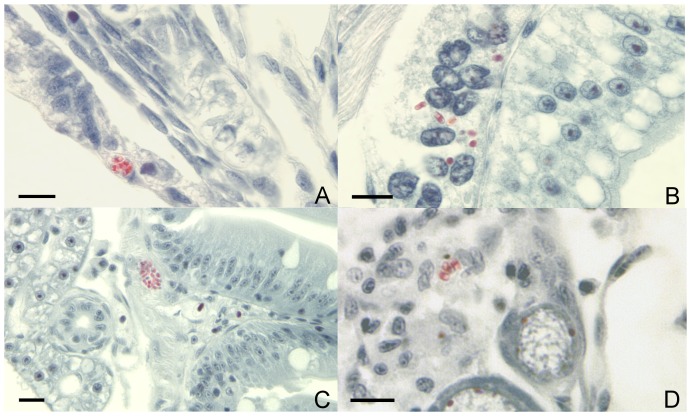
Spores of *Pseudoloma neurophilia* in Luna-stained histological sections of progeny of infected zebrafish, *Danio rerio*. **A.**Spores (red) in the epidermis of a 7 d post-fertilization (pf) larval zebrafish. **B.** Spores in the resorbing yolk-sac of the same 7 dpf larval zebrafish. **C.** Spore aggregate beneath the intestinal epithelium of an 8 wk pf juvenile fish. **D.** Spores in the ovigerous stroma adjacent to developing follicles in an 8 wk pf fish. Bar  = 10 µm.

One of 24 egg pools was positive for *P. neurophilia* DNA. Histological examination of 7 dpf larval fish revealed the presence of microsporidian spores in the epidermis (Figure 1A) and resorbing yolk sac (Figure 1B) of one of 112 fish examined. Histological examination of juveniles from this spawn, reared in isolation, at 8 wk post fertilization revealed spores in various tissues in 3 of 36 fish examined, such as the lamina propria of the intestine (Figure 1C), the ovigerous stroma (Figure 1D), and the inner ear.

**Figure 2:**
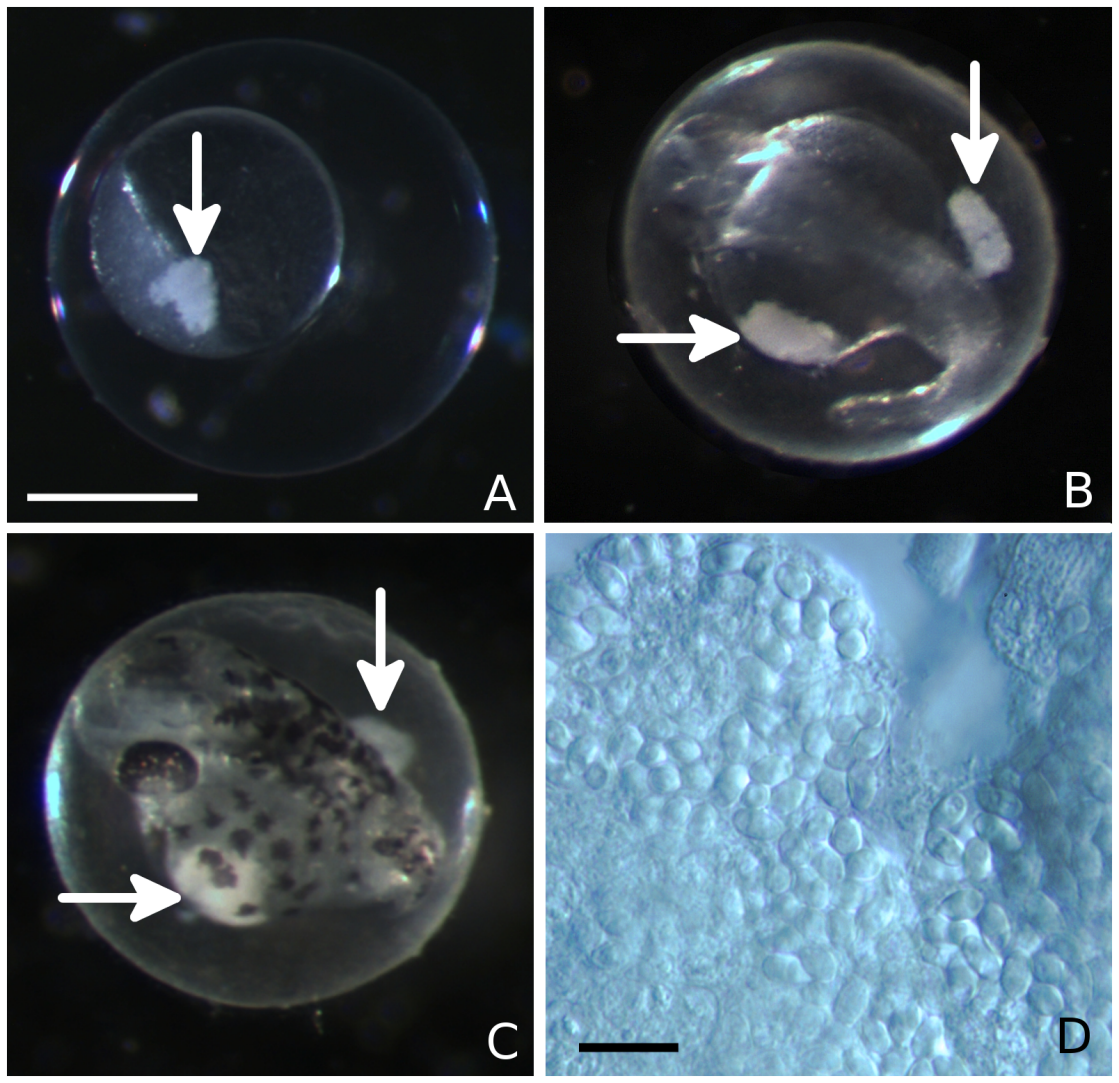
Spores of *Pseudoloma neurophilia* in developing embryo of zebrafish, *Danio rerio*. **A.** Aggregated spores (arrow) in a 4 hpf embryo. Bar  = 0.5 mm. **B.** Two foci of spores (arrows) visible in the same embryo at 24 hpf. **C.** Spores (arrows) in the same embryo at 48 hpf. **D.** Differential interference contrast micrograph of spores from an embryo. Bar  = 10 µm.

Eggs from a second group spawn of 30 adults from a population of infected fish were examined using a stereomicroscope with a transmitted light source. Distinctive opaque regions were seen in two embryos at approximately 4 hours post-fertilization (Figure 2A). One embryo was maintained at 28°C and examined again at 24 and 48 hours post fertilization during which time it appeared to develop normally (Figures 2B, 2C). The other embryo was sacrificed and examined by wet mount microscopy. Upon examination using higher magnification, these regions were found to consist of large aggregates of refractile spores with characteristic polar vacuoles, consistent in shape and size to *Pseudoloma neurophilia* (Figure 2D). The embryo was then homogenized in sterile water and the spores quantified by hemocytometer. Thirty thousand spores were present in this particular embryo.

### Prevalence of *P. neurophilia* within oocytes and detection in spawning water

*Pseudoloma neurophilia* DNA was detected within eggs from 4, and in the spawn water of 11, of 27 paired spawns (Table 1). The mean prevalence of *P. neurophilia* within spawned eggs from these 4 positive spawns was calculated to be 0.9% (CI 0.06–3.45%).

**Table 1:** Detection of *Pseudoloma neurophilia* in egg pools obtained from paired spawning of *Danio rerio* adults and tested by qPCR and in spawning adult fish by histology.

Spawn	Total eggs	Pool Size	Number positive pools/Total pools	Number of spores detected in positive egg pools	Estimated percent prevalence (95% confidence intervals)	Spores detected (water)	Histology - Male	Histology - Female
1	64	10	0/7	-	-	12159	ND	S
2	445	30	0/15	-	-	ND	ND	ND
3	543	30	0/19	-	-	16788	ND	S, F
**4**	**463**	**30**	**1/16**	**132560**	**0.2 (0.01–1.0)**	**ND**	**ND**	**S**
**5**	**151**	**30**	**2/5**	**430847, 568**	**1.3 (0.2–4.0)**	**1418**	**ND**	**S**
6	107	20	0/6	-	-	4417	S, K	S
7	223	20	0/12	-	-	1858	S	S
8	185	30	0/10	-	-	12642	S	S, O
9	135	20	0/7	-	-	ND	ND	S
10	159	20	0/8	-	-	1180	S	ND
11	295	30	0/11	-	-	1385	S	S, F
12	323	30	0/12	-	-	977	S	S
13	72	30	0/3	-	-	1549	S, K	S, K
14	249	30	0/9	-	-	ND	S	ND
15	247	30	0/9	-	-	703	S	S, I
16	370	20	0/18	-	-	ND	S, K	S
17	232	15	0/15	-	-	ND	S	ND
18	223	30	0/8	-	-	ND	S	S
19	285	30	0/10	-	-	ND	ND	S
20	193	30	0/7	-	-	ND	S, K	S, P
21	177	30	0/6	-	-	ND	S, K	S, O
**22**	**76**	**15**	**1/6**	**998**	**1.5 (0.01–6.3)**	**ND**	**S, K**	**S, O**
23	90	30	0/4	-	-	ND	S, K	S
**24**	**188**	**30**	**1/7**	**22901**	**0.6 (0.03–2.5)**	**ND**	**S, K**	**S, K, O**
25	178	20	0/9	-	-	ND	S, P	S, K
26	154	15	0/10	-	-	ND	S, K	S
27	398	30	0/14	-	-	ND	S	S,O

Estimation of prevalence of *P. neurophilia* in individual populations of eggs obtained by paired spawning of zebrafish. Prevalence is calculated using the maximum likelihood estimation method of Williams and Moffit [59]. ND =  None detected, S =  spinal, F =  follicle, I =  intestinal epithelium, K =  kidney, O =  ovigerous stroma, P =  pancreas.

### Histological examination of spawning pairs

Spores were observed in the spinal cords and brains of 43 out of 54 spawning adult fish that were examined. The parasite was observed in the ovigerous stroma of 7 of 27 females, and within developing follicles of 4 of these 27 fish (Table 1). Of the 4 females with eggs that tested positive by qPCR after spawning, spores were observed in the ovigerous stroma of two and within a developing oocyte of one. No spores were observed in the testes of any male fish examined nor in any tissues of fish in which spores were not also observed in the spinal cord.

### Dose response of larval fish to *P. neurophilia*

Larval fish in two trials became infected when exposed to 300 or 500 spores/ml, but not at lower concentrations (Table 2). Whereas there was variability between replicates and trials, owing primarily to the high numbers of mortalities observed during the duration of the experiment, there was a trend for a dose response.

**Table 2:** Dose response of larval zebrafish exposed to low numbers of *Pseudoloma neurophilia*.

	500 sp/ml	300 sp/ml	100 sp/ml	50 sp/ml
**Trial 1**				
**A**	5/11	1/14	0/8	0/6
**B**	2/10	0/9	0/5	0/3
**C**	6/20	1/11	0/5	0/6
**Total percent infected**	**31.7**	**5.9**	**0.0**	**0.0**
**Trial 2**				
**A**	0/3	1/2	0/4	0/1
**B**	4/6	1/4	0/4	0/5
**C**	2/5	1/2	0/1	0/3
**Total percent infected**	**42.9**	**37.5**	**0.0**	**0.0**

Two trials were performed in which three replicates of larval zebrafish were exposed to low concentrations of *P. neurophilia* spores. Fish surviving to seven day post exposure were euthanized and examined by microscopy for the presence of *P. neurophilia* spores.

## Discussion

A combination of indirect (qPCR) and direct (microscopy) methods demonstrated intraovum transmission of the microsporidian parasite, *Pseudoloma neurophilia*, in its zebrafish host. Evidence for the vertical transmission of fish pathogens has often been indirect or circumstantial, especially for those that can be transmitted both horizontally and vertically. Determination of whether vertical transmission occurs, and by which mechanism (i.e., intraovum versus extraovum) can be complicated by several factors including low overall prevalence of vertical transmission, variation of occurrence both within and between clutches of eggs, and the ability of many of these pathogens to survive for long periods in the water leading to extraovum transmission. Here we verify for the first time intraovum transmission of microsporidium infecting a vertebrate host.

There are several similarities and some differences between *P. neurophilia* and *Ovipleistophora ovariae*. The latter microsporidium was first described in ovarian infections of the golden shiner, *Notemigonus crysoleucas*, another cyprinid fish that is an important bait fish raised in aquaculture in the United States. It is found with high prevalence in females from commercial fish farms and has been shown to greatly reduce fecundity as the fish ages. *Ovipleistophora ovariae* is found primarily in the ovigerous stroma and within intermediate to fully mature oocytes. However, it has also been observed in liver and kidney tissue from a few infected females [31]. It has not been observed in tissue from male fish, however DNA from the parasite has been detected in testes [32]. Horizontal transmission of *O. ovariae* has been demonstrated by feeding fry and fingerlings spores adsorbed to feed [33]. By treating eggs from infected shiners with RNase Away, as done in our study, Phelps and Goodwin (2008) detected high numbers of copies of *O. ovariae* DNA in all pools of eggs tested, providing strong evidence of intraovum transmission of the parasite. Fry hatched from the same clutch of eggs also showed high levels of *O. ovariae* DNA at 48 hours post hatch. However, no parasites were observed in these fish by microscopy. The parasite almost completely replaces the egg interior with most infected eggs, and thus Summerfelt (1972) suggested that embryos from these eggs are not viable, but rather serve as a source of infection to siblings. In this case, therefore, *O. ovariae* is actually transmitted by extraovum maternal transmission because the infected egg itself does not directly result in an infected fish.

In contrast to *O. ovariae*, relatively few zebrafish eggs and embryos are infected by *P. neurophilia*. We were able to observe spores of *Pseudoloma neurophilia* in one 7 day post fertilization fish (i.e. 5 day post-hatch) by histology, and this low prevalence correlates with that found by screening eggs by qPCR (∼1%). While we are unable to differentiate between merogonic or spore stages of *P. neurophilia* using the qPCR assay, the observation of mature spores within a developing embryo and developing oocytes confirms the presence of this stage in at least some cases of intraovum transmission. This does not preclude the presence of presporogonic stages within oocytes, which are more difficult to visualize by standard histological methods. The other member of the genus *Ovipleistophora, O. mirandellae*, also infects ovaries, testis, and eggs of several fishes [19], [34]. It has been suggested to be vertically transmitted, but we are not aware of empirical data supporting this hypothesis.

There are other examples of fish pathogens that, like *P. neurophilia*, are transmitted both horizontally and vertically, and show a low prevalence of infected eggs. It is not necessary to have a high prevalence of infected eggs and embryos within a clutch to infect the next generation as vertical transmission is followed by robust horizontal transmission within the F1 siblings. Two bacterial and one viral pathogen of salmonid fishes use these modes of transmission. *Renibacterium salmoninarum*, for example, is an obligate Gram positive bacterial pathogen that has been spread around the world with egg shipments [35]. It was present within surface disinfected eggs from experimentally infected rainbow trout *Onchorynchus mykiss* at a prevalence of 1.7% [36], and from two heavily infected coho salmon *Oncorhynchus kisutch* from separate studies at prevalences of 5.8% [37] and 11.6% [38]. Similarly, 13% of newly spawned eggs from infected steelhead trout, *Onchorynchus mykiss*, were found to be infected with *Flavobacterium psychrophilum* [39], a serious pathogen in salmon and trout hatcheries [40]. The small size of these bacteria allow for their passage into the egg after it is released from the female through the egg micropyle prior to fertilization, whereas the larger size of microsporidian spores such as *P. neurophilia* (approximately 3.5 µm by 5 µm) prevents it from entering through the approximately 1.7–2.5 µm diameter micropyle of the developed zebrafish egg [41], requiring it to be present in the oocyte prior to spawning. This could account for the somewhat higher prevalence of intraovum transmission observed with these bacteria.

Infectious hematopoietic necrosis virus (IHNV), a virus infecting salmonids, was responsible for the failure of successful culture of sockeye salmon, *Oncorhynchus nerka*, in Alaska prior to 1981 [42]. The implementation of a risk management approach consisting of the use of virus-free water supplies, rigorous disinfection, and compartmentalization of eggs and fry to contain virus outbreaks when they occur was unsuccessful in completely resolving IHNV epizootics [43]. One possibility for this failure was suggested to be that intraovum vertical transmission of the virus occurs, resulting in a few subclinically infected progeny. There has been evidence to support the intraovum transmission of IHNV. Mulcahy and Pascho [44] described the isolation of IHN virus from fry and eggs held in the laboratory under virus-free conditions in two occurrences. They found that only a small proportion of eggs and fry tested contained IHN from one female with a high titer present in the body cavity.

The low prevalence of intraovum transmission of *P. neurophilia* observed is adequate to maintain the infection between generations, given the large numbers of larval siblings in a single spawn and the high rates of horizontal transmission. Indeed, observing the infection by histology, we detected *P. neurophilia* in only one 7 dpf larva, but within 8 weeks increased to 11%, including spores developing within the ovigerous stroma of one fish. Following initial infection, the parasite employs several routes of transmission [25]. Waterborne transmission occurs by co-habitation with infected fish and by ingestion of infected tissue. Zebrafish spawn every few weeks, releasing spores and infected eggs into the water which are then fed upon by tanks mates. Also, feeding on infected carcasses is an efficient route of transmission. Zebrafish do not generally attack other fish in an aquarium or school, but quickly scavenge dead fish.

In addition to intraovum transmission, *Pseudoloma neurophilia*, as well as IHN and *Renibacterium salmoninarum*, employs a second maternal transmission mechanism; extraovum transmission. High levels of *P. neurophilia* in the ovaries and in spawn water have been reported previously [25], [29], and with our paired spawn analysis the spawn water contained the parasite more often than eggs. Also, the dose response experiment agreed with the previously studies [45] showing that larvae, which begin feeding about 4–5 d after fertilization, are very susceptible. Again this is similar to *Renibacterium salmoninarum*, which is often found at high prevalence and concentration in the ovarian fluid from infected female salmon [46]. Whereas very few eggs were internally infected by this bacterium, it was detected on the surface of 38% of the eggs [36]. This strategy of extraovum transmission likely occurs with the IHN virus as well, and extends to infecting the next generation beyond the infected parents own progeny in a given river or stream [47], [48]. Traxler et al. [47] showed no correlation between infection levels in ovarian fluid compared to eggs, indicating that extraovum transmission is the major route of vertical transmission for this viral pathogen.

The mode of vertical transmission we report has practical implications. Extraovum transmission in fish can be mitigated by the use of rigorous surface decontamination of eggs whereas intraovum transmission (i.e., pathogens present within the egg) would render any surface decontamination method ineffective. This approach has been used to avoid extraovum viruses and bacteria following spawning with salmonids [49], [50], but is ineffective for *P. neurophilia* [45]. The high prevalence of *P. neurophilia* in laboratory zebrafish colonies, the risk of intraovum transmission, and the lack of disinfectants that will kill spores but not eggs [45] led to the development of a zebrafish colony which is specific pathogen free (SPF) for *P. neurophilia* [30], accomplished by rigorous screening of brood fish and progeny for the pathogen with our PCR tests [29], [51].

The population of *P. neurophilia* used in this study has been maintained for >4 years by horizontal passaging in zebrafish held in aquaria (i.e., exposing fish to infected spinal material, cohabitation with infected fish). This may have resulted in the selection of parasite strains which are most efficiently transmitted horizontally, resulting in the very low prevalence of intraovum transmission observed in the current study. Intraovum transmission does occur nonetheless, and the occurrence of this method of transmission could likely be experimentally manipulated. Several species of Microsporidia are transmitted both horizontally and vertically [4], [52]. Even among species which are transmitted solely transovarially, transmission efficiency has been observed to be variable between broods [52].

The zebrafish has been used to study host-pathogen interactions of numerous bacterial and viral pathogens [53]–[55]. *Pseudoloma neurophilia* infections of zebrafish would provide a good model to investigate the evolution of vertical transmission as it employs both horizontal and vertical transmission.

**Figure 3:**
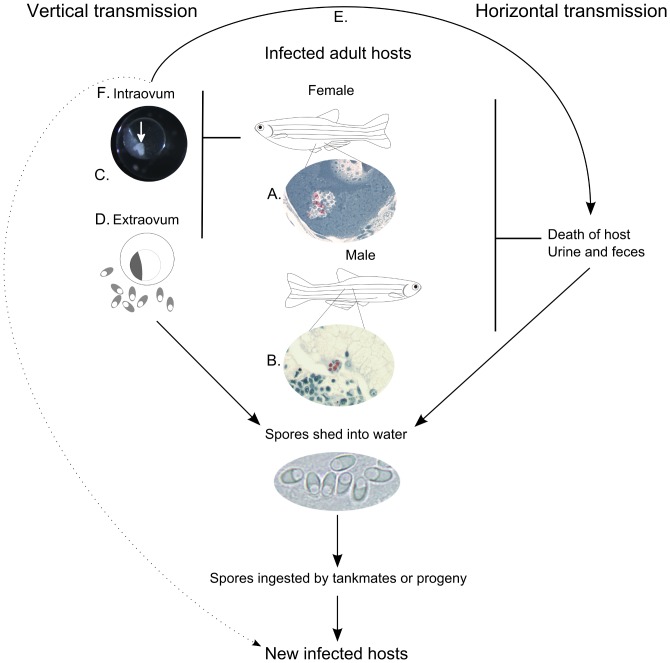
Modes of transmission of *Pseudoloma neurophilia* in the zebrafish, *Danio rerio*. **A.** Luna-stained histological section showing *P. neurophilia* spores (red) within a secondary oocyte. Sexually mature female fish have been shown to harbor the parasite in both ovigerous stromal tissue and within various developmental stages of oocytes. **B.** Luna-stained histological section of kidney from an adult male zebrafish with spores present (red) within the epithelium of a renal tubule. The presence of spores in these structures is proposed to be one method by which spores can be released into the environment by live fish. **C.** *P. neurophilia* spores (arrow) present within a developing embryo. **D.** The presence of high numbers of *P. neurophilia* spores in spawn water and the high susceptibility of larval fish can result in infected progeny. **E.** Intraovum transmission of *P. neurophilia* is proposed to result in either the death of the developing embryo or larvae with the subsequent release of spores into the water infecting tank mates or **F.** live, infected animals that then go on to transmit the parasite horizontally and, later, vertically.

We summarize the transmission of *P. neurophilia* in Figure 3: The primary mode of transmission appears to be horizontal, with many numbers of *P. neurophilia* spores being released into the water from ovaries of females during spawning (Fig. 3a), which occurs on a routine basis every few weeks. Feces and urine are also a source of infection from live fish. This is supported by the presence of *P. neurophilia* spores developing in the renal tubules of (Fig. 3b). Zebrafish are relatively docile fish, but quickly scavenge dead tank mates, providing another route of horizontal transmission.

Alternatively, vertical transmission, either intraovum (Fig. 3c) or extraovum (Fig. 3d) can occur via the female host. Spores shed by females in the ovarian fluid during spawning can then be ingested by the developing larvae, leading to new infections. Intraovum transmission can lead to death of the infected embryos (Fig. 3e) or directly to viable, infected hosts (Fig. 3f) which can subsequently infect other hosts by horizontal transmission and later, by vertical transmission. Whereas the occurrence of intraovum transmission appears to be rare, it is nonetheless important for maintenance of the parasite in laboratory zebrafish colonies especially considering that infected embryos appear to be able to develop normally, at least to the post-hatch larval stage.

## Methods

All experimental protocols for the procedures using zebrafish were approved by the Oregon State University Institutional Animal Care and Use Committee (Proposal Number: 4148).

### Dose response in larval fish

Larval zebrafish have previously been shown to be highly susceptible to *Pseudoloma neurophilia*, with an estimated 310 spores/fish exposure resulting in 2 of 6 fish becoming infected at 7 days post exposure [45]. In order to further assess the potential for extraovum transmission of *P. neurophilia* from spawned ovarian fluid to progeny, 5 dpf larval zebrafish were exposed to low numbers of the parasite and examined by wet mount for the presence of microsporidian spores developing within tissues. Two separate dose-response trials were conducted. Zebrafish embryos (AB line) were obtained from the *P. neurophilia* specific pathogen free zebrafish colony housed at the Sinnhuber Aquatic Research Laboratory at Oregon State University [30]. Embryos were placed in sterilized 100 ml glass beakers (a total of 6 beakers) in groups of 25 embryos/beaker and held in 25 ml of autoclaved system water at 28°C. At 4 dpf, larval fish were fed 500 µl of a concentrated paramecium suspension and four groups were inoculated with a suspension of *P.neurophilia* spores obtained from infected adult fish at a concentration of 300 spores/ml. At 7 days post exposure, all fish were euthanized by an overdose of Finquel (MS-222, Argent Laboratories, Redmond, WA, USA) and examined microscopically by wet mount. The presence of spores was recorded for individual fish.

### Infections in progeny from group spawns

Whereas the microsporidium has been observed in eggs at various stages of development and detected by PCR in eggs after spawning [29], it has not been visualized in embryos prior to hatching. Therefore, to obtain large numbers of potentially infected eggs, we harvested eggs that were obtained using group spawning from a known population of infected fish.

One population of presumed infected fish was spawned (approximately 75 fish) in a 10 L tank with a nylon mesh false bottom insert. Fish were held at 28°C overnight. Eggs were collected (approximately 2000), rinsed with sterile system water, placed in 90 mm glass petri dishes (approximately 200 eggs/dish) and held at 28°C in sterile embryo media [56]. At 2.5 dpf, 24 pools of ten eggs each were collected and placed in a 1.5 ml microcentrifuge tube. DNA was extracted from the egg pools and then tested by qPCR using the method described in Sanders & Kent (2011).

At 7 dpf, 112 fish were euthanized by an overdose of Finquel and fixed overnight in Dietrich's fixative. Fish were then placed in a 4X6 agarose array [57], topped with molten agarose and processed for histology. Fifteen serial 5 µm sections were cut from each array, stained with the Luna stain [58], and examined by light microscopy. At 8 weeks old, the remaining fish were euthanized and fixed in Dietrich's fixative. Thirty-six fish were processed for histology, stained with the Luna stain and examined as described above.

A second population of 30 infected adult fish was spawned. Eggs were examined for the presence of opaque regions potentially indicative of aggregates of microsporidian spores using a stereomicroscope with a transmitted light source.

### Infections in eggs and spawn water from fish spawned in pairs

In order to determine the prevalence of *P. neurophilia* present within eggs after spawning, we applied the method described by Phelps and Goodwin (2008) to determine the prevalence of *Ovipleistophora ovariae* within golden shiner eggs. Adult zebrafish that were previously exposed to *P. neurophilia* by feeding infected material were spawned in pairs in 1 L of water in a tank with a false-bottom insert. Successfully spawning pairs were euthanized by an overdose of Finquel and fixed in Dietrich's fixative for subsequent processing and histological examination. Single sagittal sections were cut from these adult fish, stained with the Luna stain, and examined for the presence of *P. neurophilia* spores. Water from the spawning tanks was collected and filtered through a 1.2 µm nitrocellulose filter and processed as previously described [29]. Eggs were collected and placed in pools ranging from 10–30 eggs, depending on the total number of eggs collected from each clutch, in 1.5 ml tubes. The commercial DNA/RNA decontaminating product RNase AWAY (Molecular BioProducts, Inc., San Diego, California, USA) was added at full concentration to pools of eggs in order to destroy any *P. neurophilia* spores or DNA present on the exterior of the eggs. After 10 minutes, the RNase AWAY was aspirated, the eggs were rinsed twice with DNA-free water and DNA was extracted from the egg pools as previously described [29] with the exception that sonication was performed in a Bioruptor sonicating bath (Diagenode, Denville, NJ, USA) at high power for 14 minutes (30 s on, 30 s off at 4°C) prior to digestion with lysis buffer.

Phelps and Goodwin [17] determined that the RNase AWAY treatment removed DNA of *Ovipleistophora ovariae* spores outside the eggs of golden shiners. To verify that this method is efficacious for *P. neurophilia* spores outside the eggs of zebrafish, we conducted the following. Eggs were obtained from a population of *P. neurophilia*-free zebrafish and placed in pools of 10 eggs in 1.5 ml tubes. Each tube was spiked with 1,000 *P. neurophilia* spores and half were treated with RNase AWAY and processed as described above with the remainder untreated. *Pseudoloma neurophilia* DNA was detected in the spiked, untreated eggs whereas no *P. neurophilia* DNA was detected by qPCR in spiked egg samples that had been treated with RNase AWAY. In order to determine whether this treatment was affecting DNA present within the egg, qPCR was performed using primers which amplify a fragment of the zebrafish *pou5f1* gene [26] on eggs which had been treated with RNase AWAY and eggs which had not. Crossing threshold (Cq) values were analyzed using Welch's t test in the statistical software package R and found to not differ significantly between RNase AWAY-treated and untreated eggs (p>0.1).

DNA extracted from egg pools and spawn water filters was analyzed by qPCR on an ABI 7500 sequence detection system using the method previously described [29]. For quantification of parasite in samples testing positive, two standard curves were obtained by spiking spawn water filters and egg pools obtained from a group spawn of known *Pseudoloma neurophilia*-free zebrafish with 100,000 *P. neurophilia* spores. Serial two-fold dilutions were made using DNA extracted from these spiked samples and the quantity of parasite was determined by plotting the Cq value of samples against the standard curve.

Estimation of *P. neurophilia* prevalence from pooled samples obtained from paired spawns was calculated using the statistical software environment R and the functions “llprevr” and “dprev” developed by Williams and Moffitt [59] and is available online (http://www.webpages.uidaho.edu/~chrisw/research/prevalence/). This method calculates pathogen prevalence using a maximum likelihood estimator based on the results of tests performed on samples consisting of variable pool sizes and provides 95% confidence intervals.
